# High-Throughput Screening Identifies Two Novel Small Molecule Enhancers of Recombinant Protein Expression

**DOI:** 10.3390/molecules25020353

**Published:** 2020-01-15

**Authors:** Jiasong Chang, Xiaoxu Chen, Ruolin Wang, Run Shi, Xiaogang Wang, Wei Lu, Sanyuan Ma, Qingyou Xia

**Affiliations:** 1 Biological Science Research Center, Southwest University, Chongqing 400716, China; jiasongchang@163.com (J.C.); chenxiaoxu95@126.com (X.C.); wangrl_1203@126.com (R.W.); shirun186@163.com (R.S.); wangyang217804@126.com (X.W.); swu_luwei@163.com (W.L.); 2 State Key Laboratory of Silkworm Genome Biology, Southwest University, Chongqing 400716, China; 3 Chongqing Key Laboratory of Sericulture, Southwest University, Chongqing 400716, China; 4 Chongqing Engineering and Technology Research Center for Novel Silk Materials, Southwest University, Chongqing 400716, China

**Keywords:** small molecules, high-throughput screening, recombinant protein

## Abstract

As a primary strategy for production of biological drugs, recombinant proteins produced by transient transfection of mammalian cells are essential for both basic research and industrial production. Here, we established a high-throughput screening platform for improving the expression levels of recombinant proteins. In total, 10,011 small molecule compounds were screened through our platform. After two rounds of screening, we identified two compounds, Apicidin and M-344, that significantly enhanced recombinant protein expression. Both of the selected compounds were histone deacetylase inhibitors, suggesting that the two small molecules increased the expression levels of recombinant proteins by promoting histone acetylation. Moreover, both molecules showed low cytotoxicity. Therefore, our findings suggest that these small molecules may have wide applications in the future.

## 1. Introduction

The use of biological drugs in the medical industry has increased dramatically worldwide, and recombinant proteins represent a dominant modality both now and in the near future [1]. Over 200 protein and peptide drugs are currently available on the market, and several hundreds more are undergoing clinical trials [2]. Most protein drugs are heterologously produced in mammalian cells to maintain structures and biochemical properties similar to those of natural proteins. Various approaches have been utilized to enhance production in mammalian cells, including using selected regulatory elements, such as strong promoters and proper signal peptides [3], optimizing gene codons [4], engineering host cells [5], and improving transfection methods [6]. Further improvements in protein expression yield are needed to reduce the time and cost of drug protein manufacturing [7].

Previous studies have demonstrated that several small molecules, such as sodium butyrate, carboxylic acids, pentanoic acid, hydroxamic acid, and valproic acid can be used to enhance protein expression in mammalian cells [8,9,10,11]. However, these molecules have limited effects on enhancement or negative effects on host cells, such as inhibiting cell growth or inducing apoptosis [12,13]. Thus, identification of novel small molecules that can enhance recombinant protein expression without negative effects on cell growth is urgently needed. Recently, high-throughput screening utilizing small molecule chemical compound libraries has been developed to identify specific drugs for diseases, inhibitors for certain biological pathways, and even inhibitors for the newly discovered gene editing protein CRISPR/Cas9 [14,15,16].

In this study, we performed a high-throughput small molecule library screen to identify novel small molecule compounds that can enhance recombinant expression in mammalian cells. Through two-round screening using a library containing 10,011 small molecules, followed by experimental verification, we identified two molecules that could improve the expression levels of recombinant proteins carried by both transiently transfected and stably integrated plasmids.

## 2. Results

### 2.1. High-Throughput Screening for Enhancing Recombinant Protein Expression

**Figure 1:**
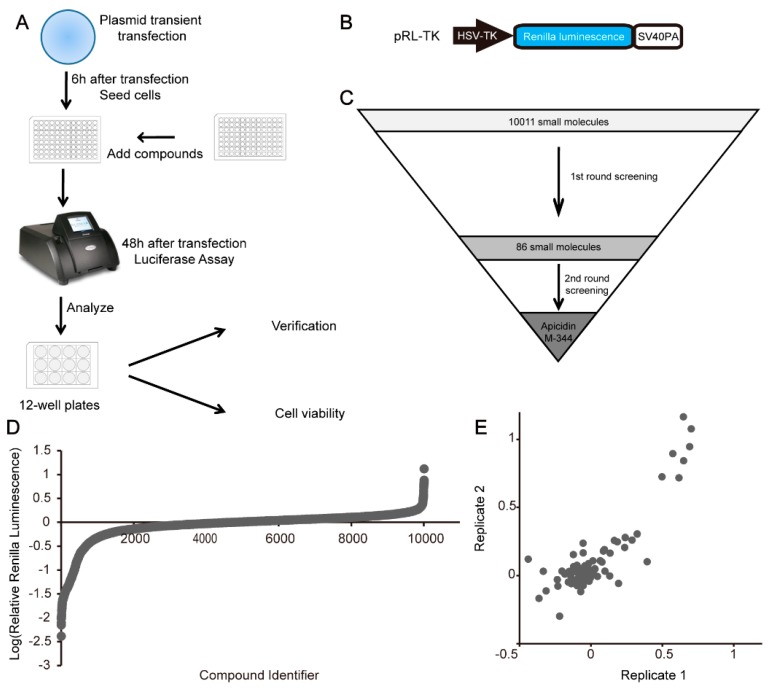
High-throughput screening for enhancing recombinant protein expression. (**A**,**C**) Schematic of the high-throughput screening platform for enhancing recombinant protein expression. (**B**) Schematic of the pRL-TK vector. HSV-TK, HSV-TK promoter; Renilla luminescence, Renilla luminescence reporter gene; SV40PA, SV40 polyA. (**D**) A waterfall plot of 100,010 compounds screened for increasing expression levels of recombinant protein. (**E**) Scatter diagram of 86 compounds evaluated in the second round of screening.

To construct the high-throughput screening platform for enhancing recombinant protein expression, we designed the strategy illustrated in Figure 1A. The Renilla luciferase expression vector pRL-TK (Figure 1B) was transiently transfected into HEK 293FT cells, and we then determined the expression levels of recombinant proteins by detecting luciferase activity. Six hours after transfection, HEK 293FT cells transfected with pRL-TK were seeded into 96-well black plates (approximately 4000 cells/well), with each well containing a known small molecule (10 μM). Then, 42 h later, Renilla luciferase activity in each well was detected using a Promega GloMax-Multi Instrument.

**Figure 2:**
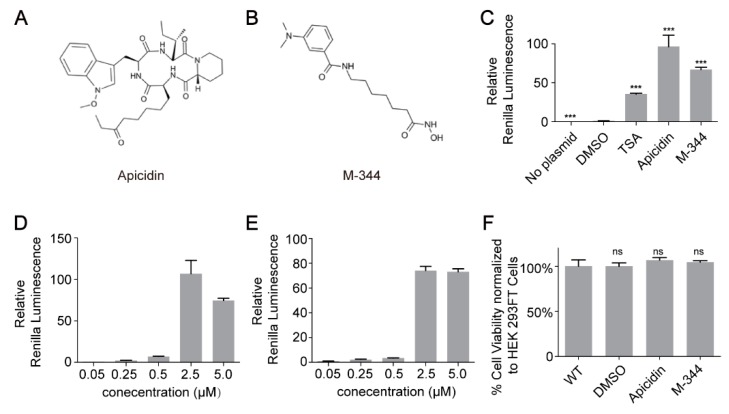
Detailed analyses of the two compounds. (**A**,**B**) The chemical structures of Apicidin (**A**) and M-344 (**B**). (**C**) Validation the improvement of Apicidin, M-344 and the positive control, TSA, using relative Renilla luminescence reporter assays. (**D**,**E**) Dose-dependent effects of Apicidin (**D**) and M-344 (**E**). (**F**) Cell viability was measured using MTS assays. All statistical analyses were determined using Student’s t-tests (ns, not significant; *** *p* < 0.001).

In total, 10,011 small molecules were selected to undergo the first round of screening (Figure 1C,D, Table S2). We identified 86 compounds that obviously enhanced Renilla luciferase activity. Then, the 86 compounds underwent the second round of screening (Figure 1C,E, Table S3). Lastly, we confirmed that the top two compounds, i.e., Apicidin and M-344, increased Renilla luciferase activity by 97- and 67-fold, respectively (Figure 2A–C). In order to investigate the magnitude of the effect of the two identified compounds, we use TSA [17] as a positive control to perform an additional transiently transfection experiment. As presented in Figure 2C, the two novel molecules show higher effects than the positive control, TSA, indicating a considerable high magnitude of the effect of the two compounds we identified. Both Apicidin and M-344 were histone deacetylase (HDAC) inhibitors (HDACIs). To evaluate the availability of the identified small molecules, the dosage effects and cytotoxicity of the two small molecules were examined. Our data showed that both small molecules had maximum effects at 2.5 μM (Figure 2D,E). We next performed 3-(4,5-dimethylthiazol-2-yl)-5-(3-carboxymethoxyphenyl)-2-(4-sulfophenyl)-2H-tetrazolium (MTS) experiments to test the cytotoxicity of the two identified compounds. The results showed that both compounds exhibited little cytotoxicity in HEK 293FT cells at the optimal concentration (Figure 2F).

### 2.2. Apicidin and M-344 Increases the Expression Levels of Recombinant Proteins

**Figure 3:**
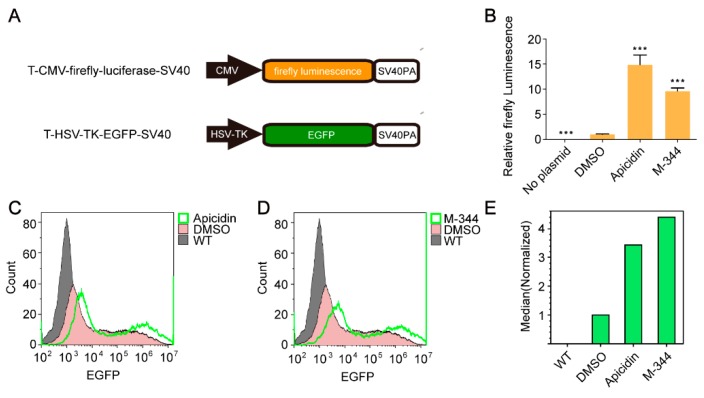
Apicidin and M-344 enhanced recombinant protein expression. (**A**) Schematic of the T-CMV-firefly-luciferase-SV40 and T-HSV-TK-EGFP-SV40 vectors. CMV, CMV promoter; firefly luminescence, firefly luminescence reporter gene; SV40PA, SV40 polyA. HSV-TK, HSV-TK promoter; *EGFP*, enhanced green fluorescent protein reporter gene. (**B**) Validation of the effects of Apicidin and M-344 using relative firefly luminescence reporter assays. (**C**–**E**) Validation of the effects of Apicidin and M-344 using flow cytometry analysis. All statistical analyses were performed using Student’s *t*-tests (*** *p* < 0.001).

To further test whether the two compounds could increase the expression levels of recombinant proteins carried by transiently transfected plasmids, a vector expressing a firefly luciferase reporter gene was constructed; this vector was designated T-CMV-firefly-luciferase-SV40 (Figure 3A). We then detected the firefly luciferase activity of HEK 293FT cells transiently transfected with T-CMV-firefly-luciferase-SV40 and treated with Apicidin or M-344. The results showed that Apicidin and M-344 increased firefly luciferase activity about 15- and 10-fold, respectively (Figure 3B). Moreover, we also constructed a vector expressing enhanced green fluorescent protein (EGFP), T-HSV-TK-EGFP-SV40 (Figure 3A), and measured green fluorescence using flow cytometry 48 h after transfection to determine whether the two compounds could enhance EGFP expression. As expected, cells treated with Apicidin or M-344 showing stronger fluorescence (Figure 3C–E). Thus, the two compounds could be used to increase the expression levels of recombinant protein during transient transfection.

### 2.3. Apicidin and M-344 Increased the Expression Levels of Genes Integrated into the Genome

**Figure 4:**
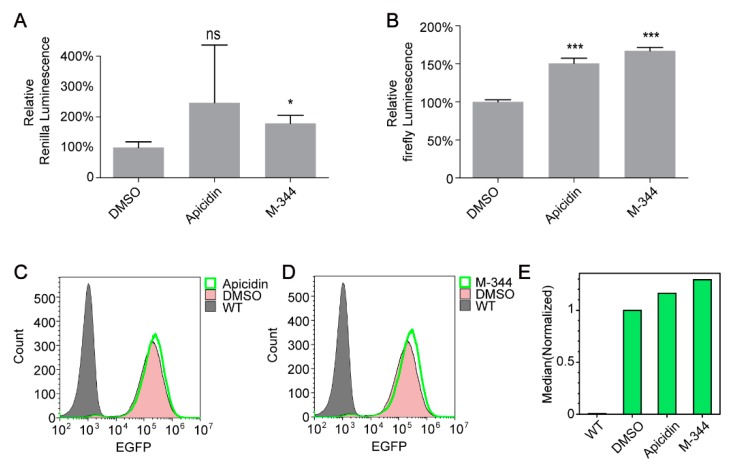
Apicidin and M-344 enhanced the expression of recombinant proteins from genes integrated into the genome. (**A**) Validation of the effects of Apicidin (**A**) and M-344 (**B**) using relative luminescence reporter assays. (**C**–**E**) Validation of the effects of Apicidin and M-344 using flow cytometry analysis. All statistical analyses were performed using Student’s t-tests (ns, not significant; * *p* < 0.05, *** *p* < 0.001).

Because histone acetylation enhances gene transcription [18], we speculated that both Apicidin and M-344 (HDACIs) would indirectly increase histone acetylation levels, thereby promoting gene expression. In order to investigate whether the two small molecules increased the expression levels of recombinant proteins from genes integrated into the genome, we performed a series of reporter gene experiments. First, we constructed a transgenic cell line with stable integration of the Renilla luciferase reporter gene and firefly luciferase reporter gene simultaneously, designated HEK 293FT-V2-34 cells. We then detected Renilla luciferase activity and firefly luciferase activity in HEK 293FT-V2-34 cells treated with Apicidin or M-344. As expected, both compounds increased the expression levels of genes integrated into the genome (Figure 4A,B). We also integrated the *EGFP* gene into the HEK 293FT genome to construct a transgenic cell line, designated HEK 293FT-CXX-4 cells, and measured green fluorescence in HEK 293FT-CXX-4 cells using flow cytometry 48 h after treatment with Apicidin or M-344. Accordingly, HEK 293FT-CXX-4 cells treated with both compounds exhibited stronger fluorescence compared with untreated control cells (Figure 4C–E).

## 3. Discussion

As major biopharmaceuticals, hundreds of recombinant proteins and peptides have been widely used in the treatment of various diseases and will play important roles in the years to come. In order to maintain correct folding for biomedical applications, recombinant proteins are primarily produced in mammalian cells through transient transfection. Because a growing number of recombinant proteins are being subjected to screening to obtain the desired biological drugs, increasing the expression levels of recombinant proteins has become an important task.

In this work, we reported a platform using luciferase as a reporter to identify novel small molecules that would increase the expression levels of recombinant proteins in mammalian cells. To this end, a small molecule compound library was used to perform the screening. Two rounds of screening were performed through our platform. In the first round, 86 potential small molecules that could enhance recombinant protein expression were identified from 10,011 small molecule compounds. Subsequently, we conducted a second round of screening. Two novel small molecule compounds, Apicidin and M-344, were shown to increase the activity of Renilla luciferase by 97- and 67-fold, respectively, at the optimal concentration. We next attempted to increase the activity of the other luciferase, firefly luciferase, using the two compounds; our results showed that the expression levels of firefly luciferase were significantly enhanced. We also used the fluorescent protein reporter system to detect the two small molecules. Flow cytometry analysis showed that both small molecules increased the expression of EGFP. Thus, our findings demonstrated that the two small molecules significantly increased the expression levels of recombinant proteins.

Although the two small molecules we selected enhanced the expression of recombinant proteins, our data showed that the degree of increase differed among various recombinant proteins. For example, in contrast to the fold changes observed for Renilla luciferase, the activity of firefly luciferase was increased by only 15- and 10-fold following treatment with Apicidin and M-344, respectively. Our data indicated that the bottleneck issues encountered by different recombinant proteins to limit expression included the DNA structure of the plasmid, codon preference, and regulatory elements of the exogenous gene. Thus, in order to increase the expression level of the target recombinant protein, it may be necessary to specifically optimize the expression plasmid.

Furthermore, we investigated the mechanisms of action of the two small molecules. Interestingly, both compounds were HDACIs. As previously reported, histone acetylation mainly occurs on lysine residues, making chromatin structure loose and promoting gene expression; however, deacetylation of histones represses gene expression [18]. Another study revealed that nucleosome-like particles could be formed on plasmid DNA after transient transfection [19]. Thus, we hypothesized that after transient transfection, the plasmids would enter the nucleus and form complexes with histones. Both of the selected compounds improved histone acetylation levels and enhanced the expression of recombinant proteins. For different transiently transfected plasmids, the DNA sequence can vary, and the post-translational modifications of histones may differ. Nevertheless, increasing histone acetylation levels may enhance recombinant protein expression overall. Our study also showed that the two small molecule compounds enhanced the expression of genes integrated into the genome, but the fold changes in expression were approximately 2, which was less than the fold change in the expression of exogenous genes carried by transiently transfected plasmids. We also tested cell activity at the optimal concentration for each compound and showed that both small molecules had little effect on cell viability.

In conclusion, we established a high-throughput screening platform for improving the expression levels of recombinant proteins. After two rounds of screening, we identified two small molecule compounds, i.e., Apicidin and M-344, that significantly enhanced the expression levels of recombinant proteins. Although HDACIs have been reported to enhance exogenous gene expression [20], the two compounds we screened may have valuable applications in the biological drug industry.

## 4. Materials and Methods 

### 4.1. Construction and Description of the Vectors

The Renilla luciferase expression vector pRL-TK (E2241) and the firefly luciferase expression vector pGL3-Enhancer (E1771) were purchased from Promega (Madison, WI, USA). *piggy*Bac{3×P3-EGFP-SV40} was from stock stored in our laboratory. The CMV promoter was amplified by polymerase chain reaction (PCR) from lentiCRISPR v2 (Addgene, cat. no. 52961) using PrimeSTAR Max DNA Polymerase (Takara, Kyoto, Japan). Additionally, the firefly luciferase reporter gene was amplified by PCR from pGL3-Enhancer using PrimeSTAR Max DNA Polymerase. These two fragments were linked together using overlap PCR to generate the CMV-firefly-luciferase-SV40 fragments and then cloned into the pEASY-T5 Zero-Complete vector (TransGen Biotech, Beijing, China) to generate T-CMV-firefly-luciferase-SV40. The HSV-TK promoter was amplified from pRL-TK, and the EGFP fragment was amplified from *piggy*Bac{3×P3-EGFP-SV40}. These two fragments were linked together using overlap PCR to generate the HSV-TK-EGFP-SV40 fragments and then cloned into PMD-19 T simple (Takara, Kyoto, Japan) to construct T-HSV-TK-EGFP-SV40. The CMV-firefly-luciferase-SV40 fragment was amplified from T-CMV-firefly-luciferase-SV40, designated DLC-1. The HSV-TK-Renilla-luciferase-SV40 fragment was amplified from pRL-TK, designated DLC-2. The EFS promoter was amplified from lentiCRISPR v2, designated DLC-3. The EGFP fragment was amplified from *piggy*Bac{3×P3-EGFP-SV40}, designated DLC-4. These four fragments were linked together using overlap PCR to generate the SYG-34 fragments and then cloned into pEASY-T5 Zero-Complete to construct T-SYG-34. Then, T-SYG-34 was cloned into the PacI/BamHI site of the lentiCRISPR v2 to generate lenti-v2-34. All restriction enzymes used in this study were purchased from NEB (Ipswich, MA, USA). All plasmids described in this manuscript are available upon request. Primers used in this study were list in the Table S1.

### 4.2. Cell Culture and Transduction

HEK 293FT cells were stored in our laboratory and cultured in Dulbecco’s modified Eagle’s medium plus GlutaMAX medium (Gibco, Waltham, MA., USA) with 10% (*v*/*v*) fetal bovine serum at 37 °C in a 5% (*v*/*v*) CO_2_ atmosphere. The culture medium was exchanged every day. All plasmids were extracted using a QIAprep Spin Miniprep Kit (Qiagen, Hilden, Germany) and transfected using Lipofectamine 2000 (Life Technologies) according to the manufacturer’s instructions.

### 4.3. Luciferase Reporter Assay

Cells transfected with luciferase reporter vectors were seeded into 96-well black plates (approximately 4000 cells/well), and each well contained a known small molecule. At 48 h after transfection, the luciferase activity was detected using the Dual-Glo luciferase assay system (Promega) with a Promega GloMax-Multi Instrument.

### 4.4. Flow Cytometry Analysis

At 48 h after transfection with T-HSV-TK-EGFP-SV40, cells were collected and washed with phosphate-buffered saline. To test the expression levels of EGFP, the cells were subjected to flow cytometry analysis using a CytoFLEX flow cytometry (Beckman Coulter, Brea, CA, USA). HEK 293FT-CXX-4 cells were subjected to flow cytometry analysis at 48 h after treatment with Apicidin or M-344.

### 4.5. Lentivirus Production and Infection to Construct a Stably Transfected Cell Line

To produce lentivirus, 20 μg lenti-v2-34, 10 μg pMD2.G (Addgene, cat. no. 12259), and 15 μg psPAX2 (Addgene, cat. no. 12260) were mixed with Lipofectamine 2000 (ThermoFisher Scientific, Waltham, MA, USA) and then transfected into HEK 293FT cells according to the manufacturer’s instructions. At 60 h after transfection, the medium was collected and purified. Then, HEK 293FT cells were infected with virus to construct HEK 293FT-V2-34 cells. HEK 293FT-CXX-4 cells were constructed using the method described above.

### 4.6. Small Molecule Compound Library

In total, 10,011 small molecules were screened. All the compounds were obtained from the National Compound Resource Center (China). The compound libraries were as follows: Kinase Inhibitor Library, Syn Kinase Inhibitors, Phosphatase Inhibitor Library, Protease Inhibitor Library, Nuclear Receptor Ligand Agonists or Antagonists Library, SIGMA LOPAC Natural Products Library, NCC-001_Shipment, ICCB Known Bioactives Library, Orphan Ligand Library, REDOX Library, ynxl2080-ncds, FDA Approved Drug Library, Epigenetics Library, Protein Kinase Inhibitor Library, Stem Cell Regulators Library, IBscreening library, Prestwick Chemical Library, TocriscreenTotal, and The Spectrum Collection.

## Supplementary Materials

The following are available online, Table S1: Primers used in this study. Table S2: The raw data of the first screen. Table S3: The raw data of the second screen.
