# Supplementary material for: High-Throughput Screening Identifies Two Novel Small Molecule Enhancers of Recombinant Protein Expression
Source: Molecules. 2020 Jan 15;25(2):353. doi: 10.3390/molecules25020353 (PMC7024190; doi:10.3390/molecules25020353)
Supplement: Supplementary file 1 [file molecules-25-00353-s001.zip › Supplementary Files/Table_S1.docx]

**Table S1. Primers used in this study**.

| Primer name | Sequence (5′→3′) |
| --- | --- |
| CMV-SSA-1F | GTCGACCCGCTCGAGACGCGTT |
| CMV-SSA-1R | CTTTATGTTTTTGGCGTCTTCCATTACGAAGACTACTAGGAGCTCTG |
| CMV-SSA-2F | CAGAGCTCCTAGTAGTCTTCGTAATGGAAGACGCCAAAAACATAAAG |
| PM-R | ATGCTAGCTACCACATTTGTAGAGGTTTTACTT |
| HSV-TK-F(AgeI) | ACCGGTAGATCTAAATGAGTCTTCGGA |
| HSV-TK-R | CTCGCCCTTGCTCACCATGGTGGCTAGCCTATAGTGAGT |
| EGFP-F | ACTCACTATAGGCTAGCCACCATGGTGAGCAAGGGCGAG |
| SV40-R | TGCAATTGGATCCATATATAGG |
| DLD-1F | TTAATTAAGGTACCCCGCTCGAGACGCGTT |
| DLC-1R | CGAGGTCCGAAGACTCATTTGGCGCGCCCGATCCGCGGTACCACATTTGTAGAGGTTTTACTT |
| DLC-2F | AAGTAAAACCTCTACAAATGTGGTACCGCGGATCGGGCGCGCCAAATGAGTCTTCGGACCTCG |
| DLD-3R | TCAAGACCTAGCTAGCGAATTCGGCGCGCCCGATCCGCGGTACCACATTTGTAGAGGTTTTACTT |
| DLD-4F | AAGTAAAACCTCTACAAATGTGGTACCGCGGATCGGGCGCGCCGAATTCGCTAGCTAGGTCTTGA |
| DLD-4R | TCGCCCTTGCTCACCATAGCGCTCTAGAACCGGTC |
| DLD-5F | GACCGGTTCTAGAGCGCTATGGTGAGCAAGGGCGA |
| DLD-5R | GGATCCGGCGCGCCTTACTTGTACAGCTCGTCCATG |
